# Design of Low-Latency Layered Normalized Minimum Sum Low-Density Parity-Check Decoding Based on Entropy Feature for NAND Flash-Memory Channel

**DOI:** 10.3390/e26090781

**Published:** 2024-09-12

**Authors:** Yingge Li, Haihua Hu

**Affiliations:** Information Engineering, Guangdong University of Technology, Guangzhou 510006, China; haihua@mail2.gdut.edu.cn

**Keywords:** entropy feature, LNMS LDPC decoding, cosine similarity, flash-memory channel

## Abstract

As high-speed big-data communications impose new requirements on storage latency, low-density parity-check (LDPC) codes have become a widely used technology in flash-memory channels. However, the iterative LDPC decoding algorithm faces a high decoding latency problem due to its mechanism based on iterative message transmission. Motivated by the unbalanced bit reliability of codeword, this paper proposes two technologies, i.e., serial entropy feature-based layered normalized min-sum (S-EFB-LNMS) decoding and parallel entropy feature-based layered normalized min-sum (P-EFB-LNMS) decoding. First, we construct an entropy feature vector that reflects the real-time bit reliability of the codeword. Then, the reliability of the output information of the layered processing unit (LPU) is evaluated by analyzing the similarity between the check matrix and the entropy feature vector. Based on this evaluation, we can dynamically allocate and schedule LPUs during the decoding iteration process, thereby optimizing the entire decoding process. Experimental results show that these techniques can significantly reduce decoding latency.

## 1. Introduction

With the rapid development of artificial intelligence, cloud storage, and hyperscale data centers, the speed of data generation and processing continues to accelerate, resulting in a sharp increase in the demand for data storage [1]. In particular, in storage technology, read latency has become a key challenge because it directly affects the response speed and processing efficiency of the system [2,3]. Optimizing the read latency of NAND flash memory channels can not only improve data access speed but also meet the needs of modern applications for high-speed data processing. At the same time, with the increase in storage density, despite the increase in storage capacity, the reliability of NAND flash memory faces challenges [4,5]. In an environment with a high bit-error rate, it becomes particularly important to use multi-threshold soft decision decoding technology, which effectively enhances error correction performance by applying multiple read voltages to achieve fine-grained memory perception, but this may also introduce higher perception latency [6,7]. In addition, the layered normalized minimum sum (LNMS) decoding algorithm has attracted widespread attention due to its high efficiency in the low-density parity-check (LDPC) decoding process. LDPC decoding first calculates the log-likelihood ratio (LLR) of each bit, and then corrects the error bit by iteratively updating the information of the check node (CN) and variable node (VN). This process inherently leads to higher decoding latency [8,9]. In each iteration of LNMS decoding, it is necessary to traverse each row of the check matrix to update the corresponding VN and CN information. The unit that processes each row during one iteration is called a layered processing unit (LPU), which is the most time-consuming step in the decoding process. Therefore, in application environments with strict real-time requirements, it is particularly important to develop an iterative decoding strategy that can dynamically adjust the average number of LPUs executed during decoding.

### 1.1. Related Work

Significant research has been conducted into the efficient application of LDPC decoding within flash memory systems. A sensing strategy to reduce the raw bit-error rate (RBER) and latency of LDPC is proposed in [10], which comprehensively considers the inter-state, intra-state and cross-layer asymmetric error characteristics of flash memory. The read voltage is optimized to reduce the RBER by utilizing the unbalanced state transition features brought on by changes in the threshold voltage distribution due to retention time and P/E cycles [11]. An optimal read voltage decision-making scheme is proposed in [12] to eliminate the read operations required for read retries, thereby reducing read latency; the scheme uses overlapping error correction codes to determine the optimal best reading voltage. In [13], the nonuniform error characteristics between different states and within the same state are comprehensively quantified, thus the sensing level and the delay can be optimized in a more targeted manner. Given the challenges posed by intra-cell unbalanced bit-error probability and data dependence, in [14], strategies such as interleaving upper and lower page bits have been proposed to enhance min-sum LDPC decoding and reduce decoding latency. A joint decoding strategy is proposed in [15] to achieve latency reduction, which optimizes soft information by exploiting the bit-granularity error rate obtained by combining two bits in a pair of shared pages. Further studies have shown that nonuniform bit-error rates across layers can be mitigated by blending data from error-prone, lower-layer units with more reliable upper-layer units, thereby diluting the concentration of errors and improving decoding speed [16]. Additionally, methods that exploit inter-state error patterns to refine the LNMS LDPC decoding process have been explored, aiming to decrease decoding latency. A bit-error-aware LDPC decoding scheme based on the bit-error characteristics of multi-level cell (MLC) NAND flash pages is proposed in [17] to reduce the number of decoding iterations. A scheme to accelerate decoding by exploiting the error patterns of 3D triple-level cell (TLC) NAND flash pages is proposed in [18], which first decodes two lower RBER pages and stores their corresponding channel LLRs and a posteriori LLRs to optimize the decoding operation of the higher RBER page. A resistance drift-aware LDPC decoding method is proposed in [19], which improves the LDPC decoding performance but also increases the writing time. An efficient LDPC coding scheme has been introduced in [20], which exploits the significant RBER difference between pages to further shorten the decoding iterations for phase-change memory. An improved sliding window decoding algorithm [21] has been proposed based on the joint source-channel coding scheme [22] for dual spatially coupled LDPC codes. Additionally, a joint-grouping shuffling scheduled decoding algorithm [23] has been introduced for the joint source-channel coding scheme in double LDPC code systems. This method considers the overall structure of the Tanner graph for both source and channel coding and applies shuffled decoding. Furthermore, an algorithm has been proposed to jointly optimize the read voltage thresholds [24] across all layers by maximizing mutual information (MMI), thereby improving soft LLR information and reducing the read latency of 3D NAND flash memory. A bilayer LDPC coding scheme [25] has been developed for MLC NAND flash memory, leveraging the inter-page asymmetry of MLC flash memory channels and storing additional parity in the lower pages.

### 1.2. Contribution

Optimizing access latency by using inter-page or inter-layer error characteristics under flash memory channels to optimize reading reference voltage or LLR information to reduce access latency usually requires sacrificing storage space to obtain additional redundant bits or increasing the latency of read operations, especially in scenarios with high-frequency read and random access, where the potential advantages of such tightly coupled read and decode operations are difficult to fully utilize.

Motivated by this problem, this paper proposes an innovative method based on the bit reliability imbalance in LDPC codewords and develops serial entropy feature-based LNMS (S-EFB-LNMS) and parallel entropy feature-based LNMS (P-EFB-LNMS) LDPC decoding algorithms based on entropy feature matrices. These algorithms aim to reduce access latency, reduce additional space usage, and decouple dense interactions between pages. First, an entropy feature vector is constructed to quantify the reliability of each bit in the codeword. Then, cosine similarity is applied to assess the reliability of the LPU. Finally, an LDPC decoding strategy based on the entropy feature vector is implemented, and its effectiveness is verified through experiments.

The main contributions of this paper are as follows:

1. An LPU reliability assessment method based on the entropy feature vector of codewords is proposed. This method provides a basis for selecting the appropriate LPU for scheduling during the iteration process.
2. Based on the reliability assessment of LPU, S-EFB-LNMS and P-EFB-LNMS LDPC decoding algorithms are proposed for serial and parallel architectures, respectively. These algorithms effectively optimize the transmission of redundant information in the decoding process by adjusting the scheduling strategy of LPU in each iteration, thereby reducing unnecessary calculation and decoding latency.
3. A comprehensive performance evaluation of the proposed algorithm is carried out, which confirms that the algorithm can significantly reduce the average number of LPUs in each iteration and the total number of LPU executed in the decoding process, significantly improving the time efficiency of the decoding process. In addition, through a detailed space overhead analysis, it is proved that the proposed algorithm effectively reduces the additional space occupation. The complexity analysis of the algorithm reveals its linear growth characteristics, indicating that the algorithm shows the advantages of efficiency and practicality when processing large-scale data sets.

The rest of this paper is organized as follows. Section 2 first elaborates on the construction of the entropy feature vector of the codeword and then presents the LPU reliability evaluation method. Section 3 introduces the basic framework of the LDPC LNMS decoding algorithm and then proposes improved S-EFB-LNMS and P-EFB-LNMS LDPC decoding algorithms. Section 4 presents the performance analysis and discussion. Section 5 concludes this paper.

***Notations:*** In this paper, the following parameters are used:

- H(v): Voltage entropy function, where *v* represents the sensing threshold voltage.
- $R_{i}$: The *i*-th read reference voltage.
- $\varepsilon_{i}$: The voltage window between $R_{i-1}$ and $R_{i}$. Specifically, $\varepsilon_{1}$ represents a voltage interval of less than $R_{1}$ and $\varepsilon_{7}$ represents a voltage interval greater than $R_{6}$.
- $LLR_{\mathrm{ch}}$: LLR obtained from the flash memory channel, where $LLR_{\mathrm{ch},j}$ represents the LLR of the *j*-th bit in a codeword.
- $f_{\mathrm{efv}}$: The entropy feature vector of a codeword, where $f_{\mathrm{efv},j}$ represents the entropy feature value of the *j*-th bit.
- $c_{\mathrm{cnp}}$: The CN processing unit constraint vector, corresponding to a row of the LDPC matrix H.
- $CS(f_{\mathrm{efv}},c_{\mathrm{cnp}})$: The cosine similarity between $f_{\mathrm{efv}}$ and $c_{\mathrm{cnp}}$.
- $CI_{ij}^{\left(l\right)}$: Information transmitted from the *i*-th CN to the *j*-th VN at the (l)-th decoding iteration, where $l=1,2,\cdots ,T_{max}$. The initial value $CI_{ij}^{\left(0\right)}$ is set to 0.
- $VI_{ij}^{\left(l\right)}$: Information transmitted from the *j*-th VN to the *i*-th CN at the (l)-th decoding iteration.
- $v^{\left(l\right)}$: Posterior information at the *l*-th decoding iteration, where $v_{j}^{\left(l\right)}$ represents the posterior information of the *j*-th bit at *l*-th iteration.
- $c_{j}^{\left(l\right)}$: The *j*-th bit of the codeword after the *l*-th decoding iteration.

## 2. Design of LPU Reliability Assessment Algorithm for Flash-Memory Systems

In this section, we first introduce the construction process of the codeword entropy feature vector in detail, including the relevant theoretical basis and implementation steps. Then, we elaborate on the LPU reliability evaluation method based on the entropy feature vector and explain how this method optimizes the selection and scheduling of LPU by analyzing the reliability information of codewords.

### 2.1. Design of Entropy Feature Vector for Flash-Memory Channel

**Figure 1:**
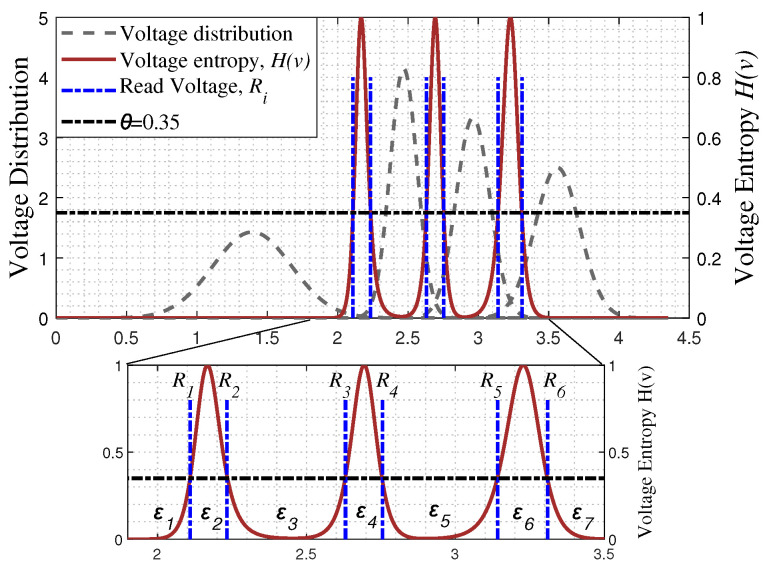
Illustration of entropy function and voltage distribution for 2-bit per cell flash-memory channel where the retention time and number of P/E cycles are set to 5000 and 20,000, respectively.

For a flash-memory cell, let *v* represent the sensing threshold voltage; its entropy can be calculated as [26]

(1) $$H\left(v\right)=-[\sum_{i}\frac{P_{s_{i}}\left(v\right)}{\sum_{i}P_{s_{i}}\left(v\right)}log_{2}\left(\frac{P_{s_{i}}\left(v\right)}{\sum_{i}P_{s_{i}}\left(v\right)}\right)],$$

where $P_{s_{i}}\left(v\right)$ represents the final threshold voltage distribution for cells of state $s_{i}$, i∈11,10,00,01. The 1-th bit is denoted as the least significant bit (LSB) and the 2-th bit is denoted as the most significant bit (MSB). An example of voltage entropy function H(v) and voltage distribution is illustrated in Figure 1. It becomes clear that entropy represents the average amount of information before and after passing through the flash-memory channel, or uncertainty. In other words, the greater the entropy, the higher the uncertainty. Then, to represent the uncertainty of the channel information of each bit for the received codeword, the entropy feature vector $f_{\mathrm{efv}}$ can be written as

(2) $$f_{\mathrm{efv}}=\left[f_{\mathrm{efv},1},f_{\mathrm{efv},2},\cdots ,f_{\mathrm{efv},j},\cdots ,f_{\mathrm{efv},n}\right],$$

where *n* is the length of the received codeword and $f_{\mathrm{efv},j}$ represents the entropy feature value of the *j*-th bit.

To reduce the overall delay caused by retrying the read operation, this paper sets the maximum number of retries for the read operation to 2 times [27], that is, using 6 soft read reference voltages. By setting the value of H(v) to 0.35 [26], one can get 6 read reference voltages, $R_{i}$, where i=1,2,3,4,5,6. Then, the voltage window is divided into 7 ranges, $\varepsilon_{1}$, $\varepsilon_{2}$, $\varepsilon_{3}$, $\varepsilon_{4}$, $\varepsilon_{5}$, $\varepsilon_{6}$, $\varepsilon_{7}$, as shown in Figure 1. According to the quantization range in which each cell falls, the mapping value corresponding to the LLR obtained from the flash memory channel (denoted as $LLR_{\mathrm{ch}}$) for a codeword in the LSB or MSB page is as shown in Table 1. Let $LLR_{\mathrm{ch},j}$ represent the LLR of the *j*-th bit in a codeword.

From Figure 1, one can observe that the entropy of the three regions $\varepsilon_{2}$, $\varepsilon_{4}$, and $\varepsilon_{6}$ is higher. Therefore, the value in the $f_{\mathrm{efv}}$ of LSB corresponding to the flash-memory cells falling within $\varepsilon_{4}$ is set to 1, and falling within other ranges is set to 0. The value in the $f_{\mathrm{efv}}$ of MSB corresponding to the flash-memory cells falling within $\varepsilon_{2}$ and $\varepsilon_{6}$ is set to 1, and falling within other ranges is set to 0, as shown in Table 2. It is instructive to note that the entropy value in the $\varepsilon_{1}$, $\varepsilon_{3}$, $\varepsilon_{5}$, and $\varepsilon_{7}$ ranges is not exactly 0, i.e., H(v)!=0. In other words, the information of a small number of cells falling into these ranges is also unreliable. Note that the mapping we have proposed in Table 2 may not be optimal, and better mappings can be found for further enhancement performance.

### 2.2. Cosine Similarity-Based LPU Reliability Assessment

Cosine similarity [28] is a measure based on the vector space model that is used to evaluate the similarity between two vectors, which is given by

(3) $$\begin{array}{cc} CS(x,y) & =\frac{x\cdot y}{∥x∥∥y∥}\\ \; & =\frac{x_{1}\times y_{1}+x_{2}\times y_{2}+\cdots +x_{n}\times y_{n}}{\sqrt{x_{1}^{2}+x_{2}^{2}+\cdots +x_{n}^{2}}\times \sqrt{y_{1}^{2}+y_{2}^{2}+\cdots +y_{n}^{2}}}, \end{array}$$

where “·” represents the dot product of vectors and “$∥\cdot ∥$” represents the length of the vector. Under certain conditions, that is, when all components of vectors x and y are non-negative, their dot product x·y is also non-negative. This is because the dot product is essentially the accumulation of the products of corresponding components, thus ensuring the non-negativity of the result. Compared with Euclidean distance, it shows unique advantages when dealing with sparse vectors, especially when most elements are zero. This feature makes it particularly suitable for sparse data scenarios, such as the processing of LDPC codes, and it can pay more precise attention to the similarity between non-zero element positions. In addition, a significant advantage of cosine similarity compared to Euclidean distance is that its value range is fixed between −1 and 1, independent of the dimension or length of the vector. This means that, no matter how long the vectors are, cosine similarity can provide a standardized similarity assessment, avoiding the changes in the metric due to increasing codeword length.

In this section, we introduce an LPU reliability assessment method based on CS. Each LPU corresponds to a row in the LDPC matrix H, which is denoted as the CN processing unit constraint vector $c_{\mathrm{cnp}}$. In $c_{\mathrm{cnp}}$, 1 indicates that the corresponding VN participates in data interaction, while 0 indicates that there is no data interaction. Then, the proposed CS-based detection algorithm aims to effectively detect the reliability of LPU by calculating the cosine similarity between $f_{\mathrm{efv}}$ (see Section 2.1) and $c_{\mathrm{cnp}}$, as follows:

(4) $$CS\left(f_{\mathrm{efv}},c_{\mathrm{cnp}}\right)=\frac{f_{\mathrm{efv}}\cdot c_{\mathrm{cnp}}}{∥f_{\mathrm{efv}}∥∥c_{\mathrm{cnp}}∥}.$$

The defining characteristic of binary LDPC matrices is that their rows consist solely of 0s and 1s. This not only underscores the sparsity of the matrix but also its distinctive structural properties. Each row in an LDPC matrix can be perceived as a vector composed of non-negative elements. Moreover, given that the entropy feature matrix also incorporates binary elements, it follows that the elements of any two such vectors, $f_{\mathrm{efv}}$ and $c_{\mathrm{cnp}}$, are non-negative. Consequently, their dot product, $f_{\mathrm{efv}}\cdot c_{\mathrm{cnp}}$, is inherently non-negative, which restrictively bounds the range of their cosine similarity ($CS(f_{\mathrm{efv}},c_{\mathrm{cnp}})$) to [0, 1]. To illustrate this, consider vectors $f_{\mathrm{efv}}=\left[0,1,0,0\right]$ and $c_{\mathrm{cnp}}=\left[1,0,1,0\right]$, where the cosine similarity is computed as follows:

$$\mathrm{CS}(f_{\mathrm{efv}},c_{\mathrm{cnp}})=0.$$

For $c_{\mathrm{cnp}}=\left[0,1,1,0\right]$, the cosine similarity is

$$\mathrm{CS}\left(f_{\mathrm{efv}},c_{\mathrm{cnp}}\right)=\frac{1}{\sqrt{2}}.$$

Further, considering the definition of $f_{\mathrm{efv}}$, it is clear that non-zero bits generally indicate unreliability in the prior information of the associated VNs. In cases where $CS(f_{\mathrm{efv}},c_{\mathrm{cnp}})$ is zero, it signifies that no unreliable node information influences the update of the corresponding CN. Conversely, when $CS(f_{\mathrm{efv}},c_{\mathrm{cnp}})$ is non-zero, it implies the involvement of unreliable verification nodes in the update process. Essentially, a higher $CS(f_{\mathrm{efv}},c_{\mathrm{cnp}})$ indicates an increase in participation by unreliable VNs, thus escalating the likelihood of violating verification constraints. Therefore, we can evaluate the reliability of the LPU output information by calculating the $CS(f_{\mathrm{efv}},c_{\mathrm{cnp}})$. The higher the $CS(f_{\mathrm{efv}},c_{\mathrm{cnp}})$, the higher the unreliability of the output information. Subsequently, we can adjust the execution strategy of the LPU during the decoding process by using the $CS(f_{\mathrm{efv}},c_{\mathrm{cnp}})$, thereby reducing the decoding delay. The proposed algorithms can be extended to support multi-level soft quantization, such as 3-level or 4-level quantization voltages. For higher-level quantization, the primary modification lies in the definition of the entropy feature matrix. In the current 2-level case, an entropy value of 1 is assigned to unreliable voltage regions, while in 3-level or 4-level quantization, additional non-zero values are assigned to different unreliable levels. The reliable voltage regions would still be assigned an entropy value of 0. Since the LDPC matrix is binary, the calculation of the cosine similarity between $f_{\mathrm{efv}}$ and $c_{\mathrm{cnp}}$ is only affected by the non-zero elements. This extension enables the proposed algorithms to handle increased noise sensitivity while maintaining their effectiveness.

## 3. Entropy Feature-Based LNMS LDPC Decoding Optimization

In this section, we first introduce the basic framework of the LNMS decoding algorithm for LDPC codes, and then we explain the working principle and key technologies of the algorithm. Then, we propose improved algorithms for serial and parallel architectures and explain in detail how these algorithms adjust their internal operations according to the reliability evaluation results of the LPU to improve decoding efficiency.

### 3.1. Generalized LNMS Decoding Algorithm

**Figure 2:**
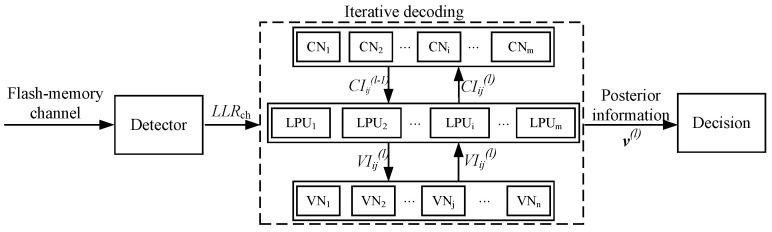
Illustration of the iterative LNMS LDPC decoding process in the receiver of a flash storage system.

This section provides a detailed description of the iterative LNMS LDPC decoding process utilized in the receiver of the flash storage system. As shown in Figure 2, the system architecture includes several key components: detector, LPU, CN, VN, and decision. The detector is mainly responsible for extracting data from the flash channel output and calculating the LLR information from the channel. This LLR information serves as the key input to the iterative decoding process and provides the necessary signal quality indicators for the subsequent steps.

In iterative decoding, the LPU plays a key role. Each LPU is responsible for processing a single row of the check matrix. For example, the *i*-th LPU mainly performs the calculation and transmission tasks of information between the *i*-th CN and the VN connected to it. At the *l*-th decoding iteration, the operation of the LPU starts by receiving external information from adjacent VNs in combination with the information transmitted to the VN by the CN in the previous round of iteration ($CI_{ij}^{\left(l-1\right)}$), where the initial value of the information transmitted by the CN to the VN ($CI_{ij}^{\left(0\right)}$) is set to zero. Then, the LPU integrates the information of all neighboring VNs and calculates and feeds back update information to the VN ($VI_{ij}^{\left(l\right)}$), thereby changing the information of the VN. In addition, the LPU is also responsible for calculating the posterior probability information of each bit and performing error detection and correction. The result of each iteration will determine the final decoding output by calculating the posterior information ($v^{\left(l\right)}$) and combining it with the decision logic. This series of iterative operations is crucial to improving the efficiency and accuracy of decoding, which continue until the preset stop condition is met or the maximum number of iterations is reached.

One can observe that, based on the principle of iterative decoding, it is obviously critical to reduce the average number of LPUs in each iteration, which directly reduces the latency of the decoding process. In addition, the optimization of resource allocation and scheduling plays a vital role in improving the operating efficiency of flash storage systems. These optimization strategies not only enhance the response speed of decoding but also improve the system’s ability to adapt to changing operating conditions, thereby promoting more efficient data processing and storage operations.

Subsequently, we briefly describe the LNMS algorithm presented in [20,29]. Let $c=\left\{c_{1},c_{2},\ldots ,c_{j},\ldots ,c_{n}\right\}$ be the received codeword from the flash-memory channel, where $c_{j}$ represents the *j*-th bit of the codeword and *n* is the length of the codeword. Let M(i) represent the set of VNs that are participated in the *i*-th CN (i=1,2,…,m). Let $CI_{ij}^{\left(l\right)}$ (i=1,2,…,m, j = 1, 2,…, n) denote the check-to-variable information at the *l*-th iteration. Let $VI_{ij}^{\left(l\right)}$ (i=1,2,…,m, j = 1, 2,…, n) denote the variable-to-check information at *l*-th iteration. Let $v_{j}^{\left(l\right)}$ (j=1,2,⋯,n) denote the posterior information at the *l*-th iteration. Let $c_{j}^{\left(l\right)}$ denote the *j*-th bit of the codeword after the *l*-th decoding iteration.

*(1) Initialization:*

Initialize the posterior information of the *j*-th bit to $LLR_{\mathrm{ch},j}$, i.e., $v_{j}^{\left(0\right)}=LLR_{\mathrm{ch},j}$. Clear the check-to-variable information, i.e., $CI_{ij}^{\left(0\right)}=0$.

*(2) Iterative decoding:*

First, at the *l*-th iteration, for the *i*-th LPU, the information of the *j*-th VN is updated with the incoming message from the *i*-th CN at the (l−1)-th iteration:

(5) $$VI_{ij}^{\left(l\right)}=v_{j}^{\left(l-1\right)}-CI_{ij}^{\left(l-1\right)}.$$

Subsequently, the information transmitted from the CN is updated with the incoming messages from its neighbor VNs, where α is the normalization factor, and it is set to 0.85 in this work. M(i)∖j represents the set of VNs participating in the *i*-th CN, except for the *j*-th VN itself.

(6) $$CI_{ij}^{\left(l\right)}=\left(\prod_{k\in M(i)\setminus j}sign\left(VI_{ik}^{\left(l\right)}\right)\right)\times \alpha \times \min_{k\in M(i)\setminus j}|VI_{ik}^{\left(l\right)}|.$$

Then, the posterior information of all bits can be calculated through

(7) $$v_{j}^{\left(l\right)}=VI_{ij}^{\left(l\right)}+CI_{ij}^{\left(l\right)}.$$

*(3) Decision:*

At the end of each decoding iteration, if $v_{j}^{\left(l\right)}>0$, $c_{j}^{\left(l\right)}=1$, otherwise $c_{j}^{\left(l\right)}=0$. If the code $c^{\left(l\right)}$ is orthogonal to **H**, i.e., $c^{\left(l\right)}\cdot H^{T}=0$, or *l* reaches the maximum iteration, the decoding iteration stops, where ${\left[\cdot \right]}^{T}$ denotes the transposition operation.

### 3.2. Serial Entropy Feature-Based LNMS (S-EFB-LNMS) LDPC Decoding Optimization Scheme

In this section, we introduce a new S-EFB-LNMS LDPC decoding method based on the similarity between the entropy feature vector and the check matrix (see Section 2.1). This decoding strategy aims to reduce the decoding delay by dynamically adjusting the number of LPUs executed during the iterative decoding process. Specifically, we evaluate the reliability of each LPU by $CS(f_{\mathrm{efv}},c_{\mathrm{cnp}})$. Each LPU corresponds to a row in the check matrix **H** and is responsible for processing the information interaction between the CN, represented by the row and the adjacent VNs. According to $f_{\mathrm{efv}}$, a value of 1 indicates that the LLR of the corresponding bit received from the channel shows high unreliability.

These LLRs are crucial as the initial information of the VNs in the decoding process. If the $CS(f_{\mathrm{efv}},c_{\mathrm{cnp}})$ of an LPU is 0, it means that the LPU does not process LLRs with high unreliability, so the output information of the LPU is considered reliable and classified as a reliable LPU (RLPU). On the contrary, LPUs with non-zero cosine similarity are marked as unreliable (URLPU). It is worth noting that, even within the voltage range defined as the reliable region, there are still voltage intervals with non-zero entropy values, which indicates that the bit LLR reliability in these intervals is low. If only the LPUs marked as unreliable are executed, the information of some unreliable VNs may not be updated, and the errors of the relevant bits cannot be corrected. To solve this problem, the interleaving parameter β is introduced to adjust the execution frequency of reliable and unreliable LPUs. For example, when β=2 is set, only RLPUs are executed in one decoding iteration, and only URLPUs are executed in the subsequent decoding iteration. If β=3, URLPUs are executed in two consecutive decoding iteration, and then reliable LPUs are executed in the third decoding iteration. This strategy effectively reduces the need to execute all LPUs in each iteration, thereby significantly reducing the decoding latency.

In addition, by monitoring and comparing the bit state changes before and after each iteration, the LPUs that will participate in the next decoding iteration are screened and optimized to improve the decoding performance further. This dynamic adjustment strategy ensures that the entropy feature vector always accurately maps the latest bit reliability data. The detailed implementation of this method has been fully described in Algorithm 1. In Algorithm 1 (line 9), the operator “%” represents the modulus (remainder) operation. It returns the remainder when dividing (l−1) by β, which is commonly used to check divisibility.
**Algorithm 1** Decoding Algorithm of S-EFB-LNMS **Input:** The LLR of one codeword from the flash-memory channel $LLR_{\mathrm{ch}}$, the entropy feature vector $f_{\mathrm{efv}}$, the maximum iteration number $T_{\max}$, and the interleaving parameter β. **Output:** Decoded bits c.  1: Initialize the posterior information of VN to the LLR from flash-memory channel, i.e., $v_{j}^{\left(0\right)}=LLR_{\mathrm{ch},j}$. Clear the check-to-variable information, i.e., $CI_{ij}^{\left(0\right)}=0$.  2: **if** $LLR_{\mathrm{ch},j}<0$ **then**  3:     $c_{j}^{\left(0\right)}=1$  4: **else**  5:     $c_{j}^{\left(0\right)}=0$  6: **end if**  7: Get RLPU and URLPU with $CS(f_{\mathrm{efv}},c_{\mathrm{cnp}})$ calculated by Equation (4).  8: **for** *l* from 1 to $T_{\max}$ **do**  9:     **if** (l−1) % β == 0 **then** 10:        Process the RLPUs. 11:     **else** 12:         Process the URLPUs. 13:     **end if** 14:     Update VN information, CN information, and posterior information calculated by Equations (5)–(7), respectively. 15:     **if** $v_{j}^{\left(l\right)}<0$ **then** 16:         $c_{j}^{\left(l\right)}=1$ 17:     **else** 18:         $c_{j}^{\left(l\right)}=0$ 19:     **end if** 20:     **if** $c^{\left(l\right)}\cdot H^{T}==0$ **then** 21:         break 22:     **else** 23:         Perform an XOR operation on $c^{\left(l\right)}$ and $c^{\left(l-1\right)}$ to find the flipped bit, and subsequently set its corresponding entropy feature value to 0 in the $f_{\mathrm{efv}}$. 24:         Refresh RLPUs and URLPUs with $CS(f_{\mathrm{efv}},c_{\mathrm{cnp}})$ calculated by Equation (4). 25:     **end if** 26: **end for**

### 3.3. Parallel Entropy Feature-Based LNMS (P-EFB-LNMS) LDPC Decoding Optimization Scheme

In Section 3.2, this paper discusses in detail the use of the entropy feature vector of codewords to optimize the serial decoding framework and reduce the frame decoding latency. Based on this, this section will focus on the development of a parallel decoding framework and its potential advantages, which is named P-EFB-LNMS LDPC decoding.

Parallel processing plays a key role in improving computational efficiency, especially in the LDPC decoding process. Using the entropy feature vector as an efficient analysis tool, this study effectively classifies the LPUs into two groups of high reliability and low reliability, namely, RLPU and URLPU, by calculating $CS(f_{\mathrm{efv}},c_{\mathrm{cnp}})$. This classification strategy ensures that cross-information interactions between VNs are avoided during the decoding process. In the simulations, we design parallel multi-threads, each independently processing LPUs of a different reliability. This parallel strategy not only ensures that both high and low reliability tasks are effectively processed but also optimizes resource allocation by processing similar tasks in a targeted manner. With this parallel design, the system can process multiple LPUs simultaneously, which significantly shortens the decoding latency and improves the overall system performance. In addition, this design also supports flexible adjustment of the workload between threads, so that it can dynamically optimize the execution strategy of threads according to the actual running performance data. The detailed implementation details and performance evaluation of the parallel processing strategy have been fully described in Algorithm 2.
**Algorithm 2** Decoding Algorithm of P-EFB-LNMS **Input:** Initial channel soft information of each bit of one frame $LLR_{\mathrm{ch}}$, the entropy feature vector $f_{\mathrm{efv}}$, the cosine similarity matrix $CS(f_{\mathrm{efv}},c_{\mathrm{cnp}})$, and the maximum iteration number $T_{\max}$. **Output:** Decoded bits c.  1: Obtain RLPUs and URLPUs by using the $CS(f_{\mathrm{efv}},c_{\mathrm{cnp}})$ calculated by Equation (4).  2: Initialize the posterior information of *j*-th bit to the LLR from flash-memory channel, i.e., $v_{j}^{\left(0\right)}=LLR_{\mathrm{ch},j}$. Clear the check-to-variable information, i.e., $CI_{ij}^{\left(0\right)}=0$.  3: **for** *l* from 1 to $T_{\max}$ **do**  4:     Process RLPUs and URLPUs in parallel.  5:     Update VN information, CN information, and posterior information calculated by Equations (5)–(7), respectively.  6:     **if** $v_{j}^{\left(l\right)}<0$ **then**  7:         $c_{j}^{\left(l\right)}=1$  8:     **else**  9:         $c_{j}^{\left(l\right)}=0$10:     **end if**11:     **if** $c^{\left(l\right)}\cdot H^{T}==0$ **then**12:         break13:     **end if**14: **end for**

## 4. Complexity and Performance

This section details the performance evaluation of the proposed S-EFB-LNMS and P-EFB-LNMS LDPC decoders by Monte-Carlo simulations, including decoding performance, decoding complexity, and space overhead, as well as the potential benefits and limitations of these techniques in practical applications.

### 4.1. Experimental Setup

In this simulations, the standard deviation and write-voltage of the erased state are set to 0.35 and 1.4, respectively, while the standard deviation of the programmed state is 0.05 and the write-voltages are 2.6, 3.2, and 3.93, respectively. For data retention noise, the constant parameters $\alpha_{i}$, $\alpha_{o}$, $A_{t}$, and $B_{t}$ are set to 0.62, 0.3, $3.5\times 10^{-5}$, and $2.35\times 10^{-4}$, respectively, where the relationship between σ and μ is set to 0.3 [30]. A 2-level soft quantization voltage is used for adjacent states. The LDPC code used is a regular code (4000, 3600) generated by the Progressive Edge Growth algorithm [31,32], with row weight $d_{c}=30$ and column weight $d_{v}=3$. The maximum number of decoding iterations is set to 15, and the number of error frames collected at each noise point is 30.

### 4.2. Performance Comparison

**Figure 3:**
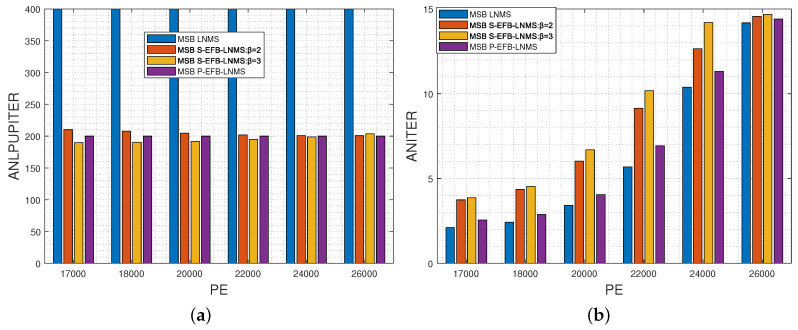
Performance comparison of LNMS, S-EFB-LNMS, and P-EFB-LNMS LDPC decoding algorithms for MSB pages: T=5000, with PE cycles ranging from 17,000 to 26,000. (**a**) Average number of layered processing units executed per Iteration (ANLPUPITER). (**b**) Average number of iterations during decoding (ANITER).

**Figure 4:**
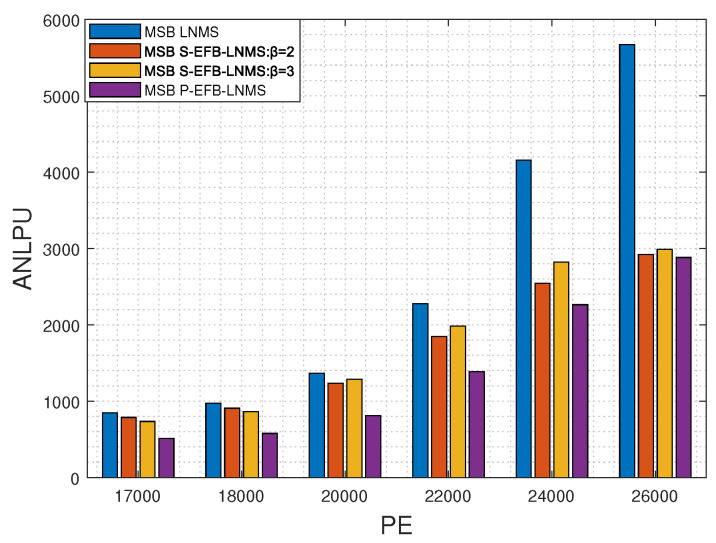
Average number of LPUs processed during decoding (ANLPU) for MSB pages using LNMS, S-EFB-LNMS, and P-EFB-LNMS LDPC decoding algorithms: T=5000, with PE cycles ranging from 17,000 to 26,000. Lower ANLPU values indicate reduced decoding latency.

**Figure 5:**
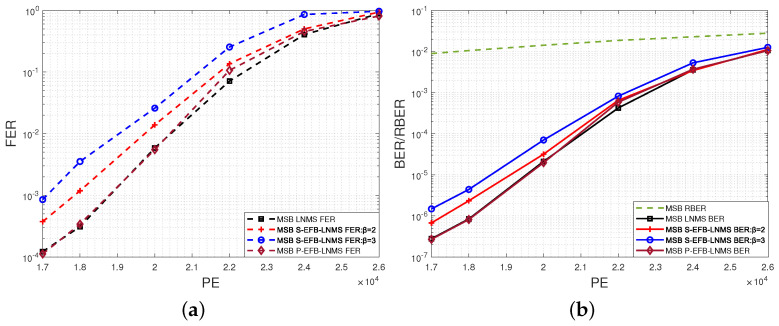
BER/FER results for MSB pages using LNMS, S-EFB-LNMS, and P-EFB-LNMS LDPC decoding algorithms: T=5000, with PE cycles ranging from 17,000 to 26,000. (**a**) FER. (**b**) BER/RBER.

This paper presents an in-depth comparative analysis of key performance indicators, including the average number of LPUs processed during decoding (ANLPU), frame error rate (FER), and bit-error rate (BER). The ANLPU is computed by multiplying the average number of iterations during decoding (ANITER) by the average number of LPUs executed per iteration (ANLPUPITER). Specific performance indicators for MSB pages, including ANLPUPITER, ANITER, ANLPU, FER, and BER, measured using LNMS, S-EFB-LNMS, and P-EFB-LNMS LDPC decoding algorithms, are presented in Figure 3, Figure 4 and Figure 5, respectively.

To verify the decoding latency advantage of the proposed algorithms, we present the results for ANITER and ANLPU. Decoding latency is determined by both the codeword length and the number of decoding iterations. When the number of decoding iterations is similar, the decoding latency is comparable [2,20,27]. For a single decoding iteration, the decoding latency primarily depends on the number of LPUs executed, where each LPU corresponds to one row of the LDPC matrix **H** in the LNMS LDPC decoding algorithm. Therefore, reducing the total number of LPUs executed during decoding can directly reduce overall decoding latency.

Figure 3a,b show ANLPUPITER and ANITER, respectively. It can be observed that by adjusting parameter β, the ANLPUPITER of the S-EFB-LNMS LDPC decoding algorithm is significantly reduced compared to the LNMS LDPC decoding algorithm [20]. This adjustment strategy proves to be effective in optimizing the processing frequency of RLPUs and URLPUs. Although the ANITER slightly increases, as shown in Figure 3b, the overall number of LPUs processed is significantly reduced, as shown in Figure 4.

Figure 4 compares the MSB page’s performance of four different algorithms (LNMS, S-EFB-LNMS:β=2, S-EFB-LNMS:β=3, and P-EFB-LNMS) in terms of ANLPU across varying PE cycles, ranging from 17,000 to 26,000. ANLPU represents the average number of layered processing units required for decoding, with lower ANLPU values indicating higher efficiency in processing overhead. Across all noise levels, LNMS consistently shows the highest ANLPU values, whereas S-EFB-LNMS and P-EFB-LNMS exhibit significantly lower ANLPU values, especially as noise levels increase. Specifically, as the noise level increases, the ANLPU value of LNMS rises from 847.6 to 5670.6, while P-EFB-LNMS only increases from 511.4 to 2881.1. S-EFB-LNMS also shows a substantial reduction in ANLPU under β=2 and β=3, with the reductions becoming more pronounced at higher noise levels.

These results demonstrate that, compared to the LNMS LDPC decoding algorithm, the S-EFB-LNMS and P-EFB-LNMS LDPC decoding algorithms both significantly improve processing efficiency, particularly under high-noise conditions, by reducing the number of processing nodes. The average reductions in the number of LPUs processed for S-EFB-LNMS with β=2, S-EFB-LNMS with β=3, and P-EFB-LNMS are 21.63%, 20.47%, and 42.49%, respectively, when compared to the LNMS LDPC decoding algorithm. Consequently, the proposed S-EFB-LNMS and P-EFB-LNMS LDPC decoding algorithms achieve a substantial reduction in decoding latency.

In addition, the P-EFB-LNMS LDPC decoding algorithm enables the simultaneous updating of the posterior probability through its unique internal design optimization by avoiding address conflicts between RLPU and URLPU. This parallel processing strategy not only significantly reduces ANLPU but also ensures high reliability (as shown in Figure 5). These improvements significantly reduce ANLPU, verifying the effectiveness of the proposed optimization strategy in shortening the average time required to decode a frame of data and increasing processing speed. Therefore, even in scenarios where the processor must execute serially, the carefully designed algorithms can effectively reduce decoding latency. In parallel execution scenarios, these algorithms not only accelerate decoding but also maintain high decoding accuracy.

**Figure 6:**
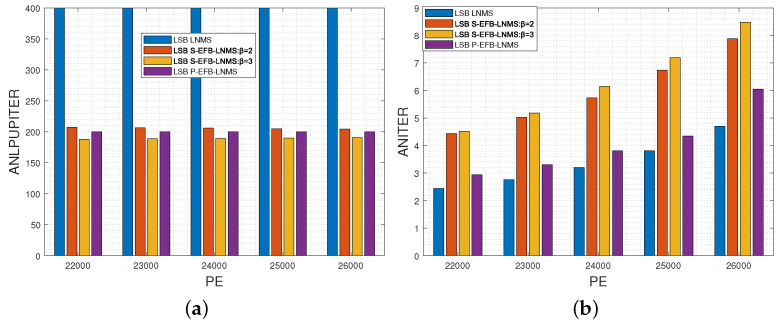
Performance comparison of LNMS, S-EFB-LNMS, and P-EFB-LNMS LDPC decoding algorithms for LSB pages: T=5000, with PE cycles ranging from 22,000 to 26,000. (**a**) Average number of layered processing units executed per iteration (ANLPUPITER). (**b**) Average number of iterations during decoding (ANITER).

**Figure 7:**
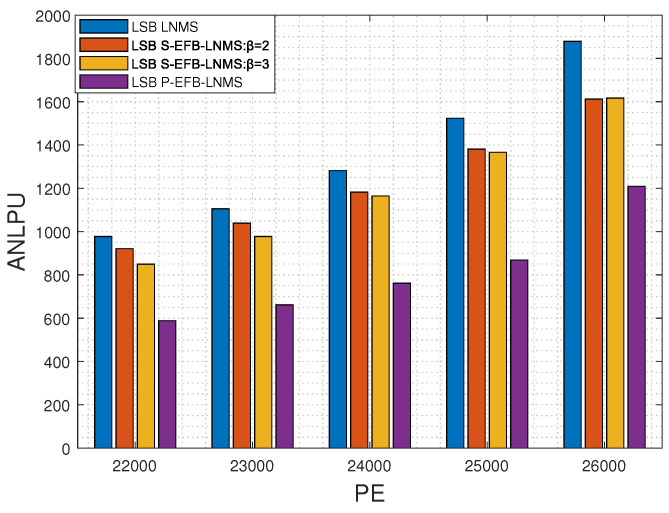
Average number of LPUs processed during decoding (ANLPU) for LSB pages using LNMS, S-EFB-LNMS, and P-EFB-LNMS LDPC decoding algorithms: T=5000, with PE cycles ranging from 22,000 to 26,000. Lower ANLPU values indicate reduced decoding latency.

**Figure 8:**
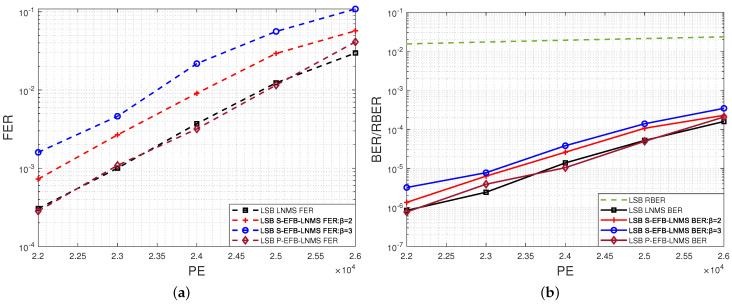
BER/FER results for LSB pages using LNMS, S-EFB-LNMS, and P-EFB-LNMS LDPC decoding algorithms: T=5000, with PE cycles ranging from 22,000 to 26,000. (**a**) FER. (**b**) BER/RBER.

Moreover, we have evaluated the performance indicators for the LSB page, including ANLPUPITER, ANITER, ANLPU, FER, and BER, as shown in Figure 6a,b; Figure 7; and Figure 8, respectively.

The performance trends for the LSB page are consistent with those observed for the MSB page, with the S-EFB-LNMS and P-EFB-LNMS algorithms demonstrating similar advantages in reducing decoding latency and improving efficiency. Overall, the proposed algorithms maintain their efficiency across both MSB and LSB pages.

Consequently, the proposed S-EFB-LNMS and P-EFB-LNMS LDPC decoding algorithms significantly reduce the average number of LPUs executed during the decoding process, thereby reducing decoding latency. These algorithms are particularly suitable for NAND flash memory applications that are sensitive to latency.

### 4.3. Computational Complexity

The computational complexity of the proposed S-EFB-LNMS LDPC decoding algorithm mainly involves the calculation of $f_{\mathrm{efv}}$ and $CS(f_{\mathrm{efv}},c_{\mathrm{cnp}})$, and the determination of RLPU and URLPU. Assume that the number of rows in the check matrix is *m* and the number of columns is *n*. The calculation of $CS(f_{\mathrm{efv}},c_{\mathrm{cnp}})$ involves the dot product of each vector and its length. Calculating the length of a vector and performing dot product operations both have a time complexity of O(*n*). Therefore, for each row in the check matrix, $CS(f_{\mathrm{efv}},c_{\mathrm{cnp}})$ is calculated, and the total time complexity is O(mn). In addition, the time complexity of updating $f_{\mathrm{efv}}$ is O(n). Based on the result of $CS(f_{\mathrm{efv}},c_{\mathrm{cnp}})$, the complexity of determining whether the LPU is RLPU or URLPU is O(m). It can be seen that the running time of the algorithm increases linearly with the increase of the number of rows and columns of the check matrix, which shows that the S-EFB-LNMS LDPC decoding algorithm is efficient and practical when processing large-scale data sets. For the proposed P-EFB-LNMS algorithm, although additional bit width is required to calculate $f_{\mathrm{efv}}$ and $CS(f_{\mathrm{efv}},c_{\mathrm{cnp}})$ during the calculation process, it is notable to point out that the calculation of $f_{\mathrm{efv}}$ and $CS(f_{\mathrm{efv}},c_{\mathrm{cnp}})$ merely impacts on the computational complexity in the design process but has no impact in the decoding process.

### 4.4. Space Overhead

The space overhead of this algorithm mainly involves the $f_{\mathrm{efv}}$ and the reliability determination results of the LPU based on $CS(f_{\mathrm{efv}},c_{\mathrm{cnp}})$. Specifically, the space requirement of $f_{\mathrm{efv}}$ is directly related to the codeword length, *n*, and its space complexity is linear. This is because each codeword bit requires 1 bit to store the corresponding entropy feature value. In addition, the classification result of each LPU also only requires 1 bit to store. Compared with the prior LLR and posterior LLR that need to store the entire page in the joint-page decoding optimization methods, the proposed method significantly reduces the storage requirement. This shows that these space overheads are all linear space complexities, indicating that, as the problem size increases, the required storage space grows linearly and controllably. This design ensures the algorithm’s high efficiency in storage resource utilization and wide applicability.

## 5. Conclusions

When dealing with the high-latency problems encountered in high-speed big-data storage systems, the LNMS LDPC decoding algorithm often exhibits insufficient decoding efficiency and response speed due to its inherent iterative processing mechanism. To overcome these limitations, we propose two improved algorithms: the S-EFB-LNMS LDPC decoding algorithm and the P-EFB-LNMS LDPC decoding algorithm. By introducing an $f_{\mathrm{efv}}$, these algorithms can more accurately locate and handle error patterns in codewords, greatly improving the overall efficiency of the decoding process. In addition, the $f_{\mathrm{efv}}$ is used in combination with the check matrix to optimize the selection and scheduling of LPUs during the iteration process. This strategy allows for more efficient use of computing resources, significantly reducing the number of required LPUs, thereby speeding up the decoding process and reducing latency. Specifically, this method ensures that each iteration makes a substantial contribution to the decoding result by reducing invalid or redundant iterations, thereby improving the overall decoding efficiency. Experimental results verify the effectiveness of these two algorithms, showing that they can significantly reduce delay in the decoding process.

## Figures and Tables

**Table 1:** Mapping between the LLR of LSB and MSB from the flash-memory channel $LLR_{\mathrm{ch},j}$ and the quantization voltage range of the corresponding cell.

	$\varepsilon_{1}$	$\varepsilon_{2}$	$\varepsilon_{3}$	$\varepsilon_{4}$	$\varepsilon_{5}$	$\varepsilon_{6}$	$\varepsilon_{7}$
$LLR_{\mathrm{ch},j}$ of LSB	−10	−10	−10	0.00001	10	10	10
$LLR_{\mathrm{ch},j}$ of MSB	−10	0.00001	10	10	10	0.00001	−10

**Table 2:** Mappings between the entropy feature value and the quantization voltage ranges of the corresponding cell.

	$\varepsilon_{1}$	$\varepsilon_{2}$	$\varepsilon_{3}$	$\varepsilon_{4}$	$\varepsilon_{5}$	$\varepsilon_{6}$	$\varepsilon_{7}$
entropy feature value of LSB	0	0	0	1	0	0	0
entropy feature value of MSB	0	1	0	0	0	1	0

## Data Availability

The data used to support the findings of this study are included within the article.
